# Adults with FPIES may face delayed diagnoses

**DOI:** 10.1016/j.jacig.2024.100304

**Published:** 2024-07-20

**Authors:** Alexandra Hua, Ian F. Slack, Kelly O’Shea, Charles F. Schuler

**Affiliations:** a Department of Internal Medicine, University of Michigan, Ann Arbor, Mich; b Division of Allergy and Clinical Immunology, Department of Internal Medicine, University of Michigan, Ann Arbor, Mich; c Mary H. Weiser Food Allergy Center, University of Michigan, Ann Arbor, Mich

**Keywords:** Food protein–induced enterocolitis syndrome, FPIES, food allergy, oral food challenge, adult FPIES

## Abstract

**Background:**

Food protein–induced enterocolitis (FPIES) is a non–IgE-mediated food allergy that is becoming increasingly recognized in adults. The time between age at symptom onset (ASO) and age at diagnosis (AD and factors affecting this gap have not been fully studied.

**Objective:**

We sought to investigate the latency between ASO and AD in adults with FPIES. We also sought to evaluate whether those patients with symptom onset in earlier years and those with comorbid gastrointestinal (GI) disease had greater mean latency.

**Methods:**

We conducted a retrospective chart review for patients with FPIES who were seen in the University of Michigan Allergy and Immunology clinic from 2015 to 2022. Patients aged 18 years and older and diagnosed with FPIES by an allergist were included (N = 19). The data collected included characteristics of the patients’ prior FPIES reactions and medical history.

**Results:**

The median age of onset of FPIES symptoms was 26 years, and the median AD was 35 years. The median difference between ASO and AD was 10 years; this difference was statistically significant according to a paired *t* test (*P* = .003). There was a negative correlation of –0.99 between year of symptom onset and latency between ASO and AD (*P* < .0001). Those patients with previously diagnosed GI conditions had a higher mean latency between ASO and AD than those without GI conditions did (*P* = .124).

**Conclusions:**

We noted a gap between ASO and AD in adults with FPIES. This gap may be due to underrecognition of adult FPIES in the past given the negative correlation with mean latency between ASO and AD. Furthermore, comorbid GI illnesses may be masking FPIES symptoms in adults, thus delaying diagnosis.

## Introduction

Although food protein–induced enterocolitis syndrome (FPIES) has been considered a primarily pediatric condition, its presentation in adults has recently been recognized.^1^ The relative novelty of the described condition may lead to diagnostic challenges for many patients. These challenges are compounded by differences in clinical presentation between adult and pediatric FPIES. Two different cohorts in Spain noted diarrhea and abdominal pain as the most common symptoms, respectively, whereas vomiting was noted less frequently.^2^^,^^3^ Culprit foods in adult FPIES differ from those in pediatric FPIES, with shellfish being the predominant culprit food in adults.^1^ Additionally, adults with FPIES may have a higher prevalence of comorbid gastrointestinal (GI) diseases.^3^ Prior studies have suggested that the current diagnostic criteria for FPIES may not be appropriate for adults.^4^

Given the lack of data on the delay in FPIES diagnoses, we sought to investigate the latency between age at symptom onset (ASO) and age at diagnosis (AD) in adults with FPIES. We hypothesized that those with symptom onset in earlier years may have greater latency because adult FPIES is a recently described condition and may have been underrecognized in the past. We also sought to evaluate whether those with previously diagnosed GI conditions had greater mean latency between ASO and AD because their symptoms might be attributed to their comorbid GI disease or inaccurately diagnosed as such.

We conducted a retrospective chart review by using data from the electronic health record for patients with FPIES who were seen in the University of Michigan Allergy and Immunology clinic from 2015 to 2022. Patients diagnosed by an allergist with *International Classification of Diseases, 10th Revision* code K52.21 for FPIES in accordance with international consensus guidelines were included.^5^ We excluded patients younger than 18 years. We recorded the characteristics of patients’ prior FPIES reactions, atopic history, and history of previously diagnosed GI diseases (including irritable bowel syndrome [IBS], ischemic colitis, and gastroenteritis).

We computed the difference between ASO and AD to determine mean latency and conducted a paired *t* test to clarify this difference. Those with unknown ASO or AD were excluded from this analysis (N = 16, 3 patients excluded from analysis). We examined 2 secondary end points of interest in relation to mean latency via Pearson correlation coefficient and ANOVA analyses, namely, year of symptom onset and previously diagnosed GI disease.

## Results and discussion

The median age of our cohort of 19 patients was 41 years; 79% (15 of 19) were female and 95% (18 of 19) had comorbid atopic conditions (Table I). The mean time to symptom onset after food ingestion was 4 hours. The median ASO of FPIES was 26 years, and the median AD was 35 years, 21% of patients (4 of 19) experiencing FPIES symptoms before age 18 years and 95% of patients (18 of 19) reacting to a single trigger. One patient underwent an FPIES oral food challenge (OFC) and reacted. All of the patients (100%) reported vomiting as a symptom, 52.6% (10 of 19) reported nausea, and 47.4% (9 of 19) reported diarrhea. Of the 19 patients, 8 (42%) had previously diagnosed IBS, 2 (11%) had acute viral gastroenteritis, 1 (5.3%) had ischemic colitis, and 4 (21%) had a severe reaction requiring an emergency room visit for intravenous hydration.

**Table I:** Patient characteristics

Characteristic (N = 19)	Value
Age (y), median (range)	41 (21-77)
Sex, no. (%)	
Female	15 (79)
Male	4 (21)
Ethnicity, no. (%)	
Non-Hispanic	17 (89)
Hispanic	1 (5.3)
Unknown	1 (5.3)
Race, no. (%)	
White	16 (84)
Asian	2 (11)
Unknown	1 (5.3)
Family history of atopy, no. (%)	9 (47)
Comorbid atopic disease, no. (%)	18 (95)
Allergic rhinitis	17 (89)
Asthma	8 (42)
Atopic dermatitis	6 (32)
IgE-mediated food allergy	4 (21)
Comorbid GI disease, no. (%)	8 (42)
IBS	5 (26)
Gastroenteritis	2 (11)
Ischemic colitis	1 (5.3)
ASO (y), median (range)	26 (7-73)
AD (y), median (range)	35 (17-73)
Latency onset vs diagnosis, median (range)	10 (<1 to 48)
No. of triggers, median (range)	1 (1 to ≥5)
Time to symptom onset (h), mean (range)	4 (1-8)
Symptoms, no. (%)	
Vomiting	19 (100)
Nausea	10 (53)
Diarrhea	9 (47)
Abdominal pain	2 (11)
Lethargy	1 (5.3)
Underwent OFC	1 (5.3)

**Fig 1:**
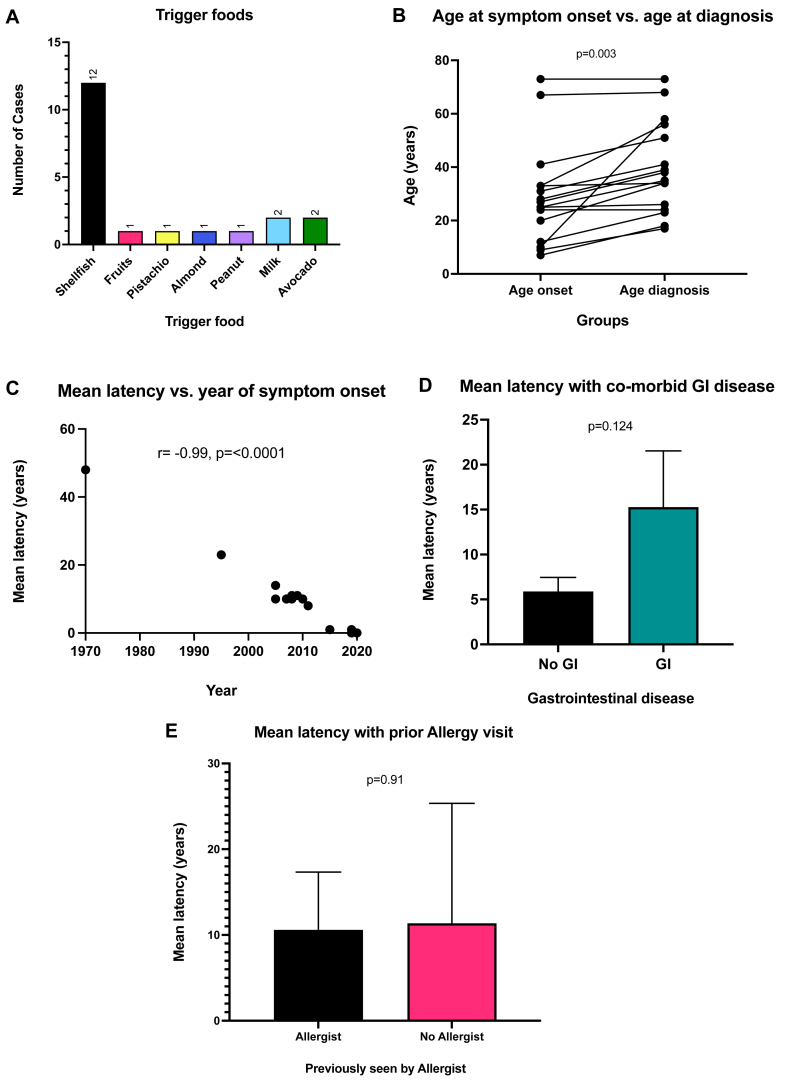
**A,** Trigger foods (N = 19 patients). One patient reacted to 2 foods. The term *Fruits* refers to 1 patient’s report of banana, blueberry, mango, papaya, and pineapple. **B,** ASO versus AD (N = 16 patients). Paired *t* test comparing the difference between ASO and AD (*P* = .003). Those with unknown ASO or AD status were excluded. **C,** Mean latency versus year of symptom onset (N = 16 patients). Correlation between year of symptom onset and mean latency between ASO and AD, as measured by using Pearson coefficient (*r* = –0.99; *P* < .0001). Those with unknown mean latency were excluded. **D,** Mean latency with comorbid GI disease (N = 16 patients). ANOVA comparing differences between means (*P* = .124). Comorbid GI conditions include IBS, ischemic colitis, and gastroenteritis. Those with unknown mean latency were excluded. **E,** Mean latency with prior allergy department visit (N = 16 patients). ANOVA comparing differences between means in those who had and those who had not seen an allergist before FPIES diagnosis (*P* = .91). Those with unknown mean latency were excluded.

The most common FPIES food trigger was shellfish (affecting 67% of patients [12 of 19]), followed by milk (affecting 11% [2 of 19]) and avocado (affecting 11% [2 of 19]) [Fig 1, *A*]. The other foods comprising the minority of food triggers included pistachio, almond, and peanut. One patient reported reactions to multiple fruits, including banana, mango, pineapple, papaya, and blueberry. The median difference between ASO and AD was 10 years; this difference was deemed statistically significant according to a paired *t* test (*P* = .003) [Fig 1, *B*]. There was a negative correlation of –0.99 between year of symptom onset and latency between ASO and AD (*P* < .0001) [Fig 1, *C*]. Those patients with previously diagnosed GI conditions had a higher mean latency between ASO and AD than those without did, although this difference was not statistically significant according to ANOVA analysis (*P* = .124) [Fig 1, *D*]. Those patients who had been seen by an allergist for other atopic conditions before their FPIES diagnosis had a mean latency between ASO and AD of 8.8 years (n = 5), whereas those who had not been previously seen by an allergist had a mean latency of 11.4 years (n = 11), although this difference was not significant [Fig 1, *E*].

The characteristics of our cohort were similar to the previously reported characteristics of adult patients with FPIES, with a female predominance and majority reacting to shellfish.^2^^,^^3^^,^^6^^,^^7^ Shellfish were the most common trigger foods for adult FPIES, as studied to date.^8^ Although the majority of patients had comorbid atopic conditions, this was likely due to the selected population of adults diagnosed in the allergy clinic. Few patients underwent OFC, which is line with previous studies, in which many adult patients decline OFC owing to prior severe symptoms.^2^^,^^4^ In contrast to the results of prior studies, all patients in our cohort reported vomiting during reactions to trigger foods and less than half reported diarrhea.^2^^,^^3^

We noted a gap between ASO and AD in adults with FPIES. This gap may be due to patients having few episodes and attributing their symptoms to other causes (ie, acute gastroenteritis) and/or providers being unaware of FPIES as a disease. We found that year of symptom onset was negatively correlated with mean latency, supporting our hypothesis that adult FPIES may have been underrecognized in the past after its initial description and before the advent of the formal *International Classification of Diseases, 10th Revision* code.^1^ Diagnostic latency has been brief for patients with symptom onset in recent years. It is important to note that our analysis was restricted to patients within a referral center allergy clinic; therefore, patients who have not or cannot access allergy care may still face significant barriers to diagnosis.

Those with previously diagnosed IBS, ischemic colitis, or acute gastroenteritis may have a greater mean latency between ASO and AD. We hypothesize that this could be due to misdiagnosis of FPIES as a GI condition or to a set of FPIES symptoms being attributed to comorbid GI disease. GI illnesses may mask FPIES symptoms in adults and delay diagnosis. Nonetheless, when those patients with and without previously diagnosed GI conditions were compared, a significant difference in latency between ASO and AD was not found. More data are needed to elucidate whether an association exists.

Limitations of our work include the small sample size, which attenuates the study’s ability to generalize to the greater population. Whether the delayed AD could be related to patients avoiding trigger foods earlier in life rather than to truly waiting to seek treatment despite symptoms is unknown. Whether providers asked specifically about each symptom of interest is not noted; thus, the reported numbers may not reflect the true prevalence of specific symptoms. The wide range of years between symptom onset and diagnosis inevitably introduces recall bias, as diagnosis was made on the basis of patients’ self-reported symptoms. Further research is needed to define the symptomatology, prevalence, and natural history of adult FPIES to better detect and treat these patients. All studies of adult FPIES to date have been conducted in relatively small cohorts with a lack of double-blinded placebo-controlled OFCs. The reasons for patients declining OFC should be investigated, as OFC remains the criterion standard for diagnosis and can be used to risk-stratify patients, subsequently guiding management. Whether adult FPIES presents in a manner clinically different from that of pediatric FPIES and whether the current diagnostic criteria are applicable to both populations remain unclear.

**Clinical implications**

A significant difference of 10 years was demonstrated between ASO and AD in adults with FPIES. Clinician awareness of the disease and patients’ GI illness may be contributing to diagnostic delay.

## Disclosure statement

This work did not receive or require direct funding. Related work was supported by the 10.13039/100007270University of Michigan via the Ronald Koenig, MD, PhD, 10.13039/100017094Department of Internal Medicine Early Career Endowment (to C.S.), the 10.13039/100001130Gerber Foundation (award 9026 [to C.S.]); and the 10.13039/100000060National Institute of Allergy and Infectious Diseases of the 10.13039/100000002National Institutes of Health (award K23AI162661 [to C.S.]).

Disclosure of potential conflict of interest: The authors declare that they have no relevant conflicts of interest.
